# Blind Image Quality Assessment Using Convolutional Neural Networks

**DOI:** 10.3390/s25227078

**Published:** 2025-11-20

**Authors:** Mariusz Frackiewicz, Henryk Palus, Wojciech Trojanowski

**Affiliations:** Department of Data Science and Engineering, Silesian University of Technology, Akademicka 16, 44-100 Gliwice, Poland

**Keywords:** image quality assessment, convolutional neural network, image database, deep learning

## Abstract

In the domain of image and multimedia processing, image quality is a critical factor, as it directly influences the performance of subsequent tasks such as compression, transmission, and content analysis. Reliable assessment of image quality is therefore essential not only for benchmarking algorithms but also for ensuring user satisfaction in real-world multimedia applications. The most advanced Blind image quality assessment (BIQA) methods are typically built upon deep learning models and rely on complex architectures that, while effective, require substantial computational resources and large-scale training datasets. This complexity can limit their scalability and practical deployment, particularly in resource-constrained environments. In this paper, we revisit a model inspired by one of the early applications of convolutional neural networks (CNNs) in BIQA and demonstrate that by leveraging recent advancements in machine learning—such as Bayesian hyperparameter optimization and widely used stochastic optimization methods (e.g., Adam)—it is possible to achieve competitive performance using a simpler, more scalable, and lightweight architecture. To evaluate the proposed approach, we conducted extensive experiments on widely used benchmark datasets, including TID2013 and KADID-10k. The results show that the proposed model achieves competitive performance while maintaining a substantially more efficient design. These findings suggest that lightweight CNN-based models, when combined with modern optimization strategies, can serve as a viable alternative to more elaborate frameworks, offering an improved balance between accuracy, efficiency, and scalability.

## 1. Introduction

The development of the Internet and significant progress in imaging device technology (cameras, smartphones, etc.) have led to the acquisition and processing of a vast number of digital images. The quality of these images is critical for the performance of vision systems. Therefore, there is a need for metrics and algorithms for image quality assessment (IQA) capable of replacing the most reliable evaluation procedures, namely subjective assessments performed by human observers and reflecting the properties of the human visual system (HVS). These assessments are typically expressed as the mean opinion score (MOS), defined as the average of individual subjective ratings. IQA methods are commonly classified into three categories: Full-Reference IQA, Reduced-Reference IQA, and No-Reference IQA [1]. The latter category is also called the Blind image quality assessment (BIQA). A reference image is understood as an undistorted image representing the original, high-quality content. Methods belonging to the FR-IQA and RR-IQA classes, which utilize reference information, generally provide strong performance [2,3]. Unfortunately, in many real-world applications, access to reference images is often unavailable. BIQA methods, which do not require access to reference images for quality assessment, are finding a growing number of applications. Rapid progress in BIQA research has been made, from NSS-based methods to deep learning (DL) models [4,5].

In the age of artificial intelligence, deep neural networks—particularly convolutional neural networks (CNNs)—have outperformed traditional approaches by enabling joint end-to-end learning of features and regression directly from raw input data [6]. In recent years, CNN architectures inspired by models such as ResNet and VGG have become less dominant in BIQA, increasingly being complemented or replaced by vision transformers, which allow models to achieve higher correlations with MOS. However, CNNs continue to play an important role in BIQA systems designed for mobile, embedded, and other resource-constrained environments, where high computational efficiency and low power consumption are required. In such scenarios, lightweight CNN architectures (typically containing 1–10 million parameters) offer fast image quality prediction. The BIQA model proposed in this paper adopts this lightweight design, providing computational efficiency while maintaining competitive performance and suitability for the aforementioned applications.

The paper is organized as follows. After a brief introduction that outlines the IQA topics, Section 2 discusses the applications of CNNs in the field of BIQA. This section references both classic works from 10 years ago and several recent review articles. In Section 3, we describe the details of the proposed network architecture and the programming tools and computer hardware used to develop CNN. Section 4 presents the experimental results obtained from the TID2013 and KADID-10k image databases. Finally, the conclusions are presented in Section 5.

## 2. Related Work

Blind image quality assessment (BIQA) methods have evolved from classical feature-based approaches, which relied on hand-crafted statistics, to CNN-based models that automatically learn hierarchical representations from images. Early CNN architectures were shallow and computationally efficient but limited in performance, while deeper networks improved accuracy at the cost of higher computational and memory demands. Recent efforts include lightweight CNNs and transformer-based approaches, which aim to balance performance with efficiency. Despite these advances, a clear research gap remains: many state-of-the-art BIQA models still exhibit high parameter counts and slow inference, highlighting the need for compact, computationally efficient architectures that maintain competitive accuracy.

The typical convolutional neural networks (CNNs) are a class of deep learning models specifically designed for processing data organized on a grid, such as images. The architecture of a CNN consists of an input layer, multiple hidden layers, and an output layer. The hidden layers include convolutional layers, pooling layers, and fully connected layers.

Convolutional layers are the core of CNNs, using filters to extract features such as edges and textures from images. Activation functions like ReLU add non-linearity. Pooling layers then reduce feature map size and complexity while keeping key information (common types: Max, Sum, Average pooling). Finally, fully connected layers use the extracted features to make predictions.

The output layer typically employs the softmax activation function for multi-class classification tasks. This function is used to generate probability values for each possible output label, with the final predicted label being the one with the highest probability score. In addition to image classification, CNNs are applied in various computer vision tasks, such as object detection in images (e.g., YOLO), image segmentation (e.g., U-Net), and face recognition (e.g., Facenet).

CNN is a class of deep learning models that are effective in processing image data, making them useful for tasks such as image quality assessment. Numerous studies have demonstrated the effectiveness of deep learning in building advanced IQA models, showing that deep neural networks can approach the functioning of the human visual system (HVS). Due to their architecture, a CNN can automatically learn key features related to image quality, thus eliminating the need for manual feature design. Different CNN models can be tailored to BIQA tasks, and through deep learning, they achieve better results than traditional image quality assessment methods. The use of CNN in BIQA also faces various challenges. Training CNNs requires large and well-labeled datasets (image bases). Training CNNs requires significant computational resources, and such models do not always perform well on new, unseen data, which is particularly important when dealing with diverse images.

The applications of CNN networks in the BIQA field began more than 10 years ago. One of the first models of this type, operating in end-to-end mode, was proposed by Kang et al. [7]. The authors trained a five-layer CNN including a convolutional layer (50 kernels of 7 × 7 pixels size), a pooling layer, two fully connected layers for feature extraction, and a linear regression layer on image patches with dimensions of 32 × 32. During the testing phase of the model, the image quality score was obtained by averaging the quality results for the individual image patches. Kang et al. also proposed a multitasking model called IQA-CNN++ in their paper [8], which simultaneously assesses image quality and identifies distortions. The proposed model uses image patches of size 32 × 32 pixels and consists of several shared layers and two task-specific layers. The shared layers included two convolutional layers, three pooling layers, and two fully connected layers with ReLU activation functions. Linear and logistic regression layers were used to predict image quality and identify distortions, respectively. Other relatively simple CNN models, similar to the networks proposed by Kang, typically consist of only one or two convolutional layers, considerably limiting their capacity for feature learning.

Several review articles on BIQA have already been published, with a particular emphasis on methods using convolutional neural networks [4,5,6,9]. The authors compare the prediction performance of different CNN models in image databases and describe some challenges in designing and training these models. Convolutional neural networks can automatically extract deep features that allow for the assessment of image quality and optimize these features to improve prediction efficiency. However, the problem lies in the lack of sufficient public image databases of distorted images along with quality assessments intended for training CNNs.

From the perspective of input to a CNN model, two approaches can be distinguished: patch input and image input. Patch input refers to a technique in which an image is divided into smaller rectangular patches (e.g., 16 × 16 or 32 × 32 pixels), which are then fed into the model as input data. This approach reduces memory usage and computational cost as the model does not need to process the entire image at once, enabling more efficient processing. It was used in the first CNN-based BIQA model [7], where quality scores predicted for individual patches were averaged to obtain the final quality estimate for the entire image. Some models select only salient patches for quality prediction [10]. The term image input indicates that the full image matrix is provided to the model without division into patches. Typical input sizes in such models include, for example, 224 × 224 pixels, as used in architectures such as ResNet or VGG.

It should be noted that in recent years there has been an increased interest in neural network architectures other than CNNs for BIQA purposes. These include vision transformers [11] and GAN-based networks [12]. In parallel with the development of advanced architectures, interest has also grown in lightweight BIQA models designed for low-resource environments, including mobile devices [13,14].

## 3. Materials and Methods

The model we propose is based on the architecture introduced by Kang et al. [7], deemed the first to apply CNNs to BIQA problems. Although current methods report higher accuracy, this solution stands out because it provides significantly lower computational cost and enhanced explainability.

### 3.1. Data Preprocessing

Data preprocessing was heavily inspired by the implementation by Kang et al. [7]: images are normalized and partitioned into non-overlapping patches. Furthermore, performance improvements were achieved through the integration of state-of-the-art techniques, which were not widely available or commonly used when the original algorithm was developed. In particular, the Tree-structured Parzen Estimator was utilized for hyperparameter tuning, and the Adam optimizer was employed for model training. The distorted images from TID2013 and KADID-10k were divided into training, validation, and test sets in proportions of 60%, 20%, and 20%, respectively. Before being fed into the model, the input data was normalized. Normalization was performed locally, meaning that the mean and standard deviation are computed within each non-overlapping neighborhood (typically a 3 × 3 window). Formally, the normalization process of the input image *I* can be described as follows:

(1) $$\hat{I}\left(x,y\right)=\frac{I(x,y)-\mu (x,y)}{\sigma (x,y)+C},$$

where

(2) $$\mu \left(x,y\right)=\sum_{i=-k}^{k}\sum_{j=-k}^{k}\omega \left(i,j\right)\;I\left(x-i,y-j\right)$$

is the local mean, (i,j) is the position of an arbitrary pixel, ω is the kernel—a 3 × 3 region,

(3) $$\sigma \left(x,y\right)=\sqrt{\sum_{i=-k}^{k}\sum_{j=-k}^{k}\omega \left(i,j\right)\;{\left(I(x-i,y-j)-\mu (x,y)\right)}^{2}}$$

is the local standard deviation, and *C* is a non-zero constant that prevents division by zero.

The normalized images are subsequently divided into non-overlapping 32 × 32 patches. Each patch is assigned the ground-truth score of the corresponding original image. This approach is justified by the homogeneity of the distortions considered, as the distortion level is consistent across all regions of the image. Furthermore, this method effectively increases the dataset and enhances the training process.

### 3.2. Proposed Architecture

**Figure 1:**
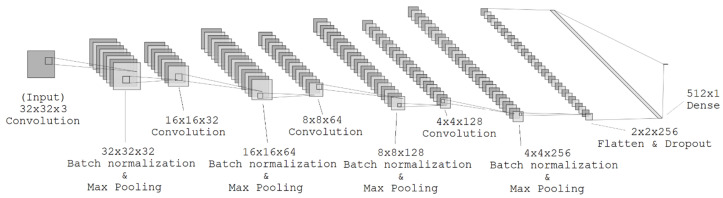
Architecture of the proposed neural network.

Figure 1 shows the architecture of the proposed neural network. The model takes as input a normalized 32×32 image patch. The input is processed by four convolutional blocks, each containing a convolutional layer, a batch normalization layer, and a max pooling layer. The details are outlined in Table 1. The number of parameters in the proposed neural network is approximately 0.9 M parameters, which qualifies the network as a lightweight neural model designed for BIQA. The computational cost is approximately 0.031 GFLOPs, which indicates that the model is computationally efficient.

ReLU was selected as the activation function for the convolutional layers and the dense (fully connected) layer. The formula for ReLU is defined as follows:

(4) $$f\left(x\right)=\max\left(0,x\right),$$

where f:R→R, is the ReLU activation function and x is the linear output of the previous layer. Mean Absolute Error (MAE) was used as the loss function to optimize model parameters. MAE is defined by the following formula:

(5) $$MAE=\frac{1}{N}\sum_{i=1}^{N}\left|{\hat{y}}_{i}-y_{i}\right|,$$

where ${\hat{y}}_{i}$ denotes *i*-th ground-truth score, $y_{i}$ is the *i*-th predicted score, and *N* is the number of training examples.

Following the last convolutional block, the output shape is 2 × 2 × 256. As the fully connected layer accepts a one-dimensional vector as input, the data needs to be reshaped. This reshape operation is known as data flattening and involves converting a three-dimensional tensor into a one-dimensional vector by concatenating all values into a single array. The flatten layer performs this operation by transforming the output shape, resulting in a vector of size 1024, which can then be fed into the dense layer. To increase generalizability and mitigate the risk of overfitting, a dropout layer is placed immediately before the final output layer. Dropout is a regularization technique that temporarily deactivates a selection of neurons during training. During a forward pass, any neuron can be dropped with a probability determined by the dropout rate, a hyperparameter. In practice, deactivation involves setting the neuron’s activation to zero.

The Adam optimizer was selected to update the model weights. Adam is widely used in training neural networks. It combines the best properties of the classical optimizers such as Momentum and RMSProp [15]. The output of the model is an estimated quality score, whose range and values vary depending on the characteristics of the input dataset. Given the number of trainable components—including four convolutional layers and a dense layer—manual selection of optimal hyperparameters becomes increasingly complex and impractical. A widely adopted state-of-the-art solution to this issue is the use of automated hyperparameter optimization.

Consequently, the Optuna framework (version 4.0.0) was used to perform this task. Specifically, the number of neurons in each layer, the learning rate, and the dropout rate were optimized. The hyperparameter search was guided by the Tree-structured Parzen Estimator (TPE) algorithm [16]. A characteristic feature of TPE is the use of decision trees to model probability distributions, in order to efficiently search for hyperparameters. This technique identifies hyperparameter combinations with a high probability of yielding better results. The hyperparameter search space used during tuning is presented in the table below.

Table 2 presents the hyperparameters included in the automated Bayesian tuning process. The first entry, n_neurons, corresponds to the number of neurons in the dense layer, followed by the learning rate and the dropout rate, respectively. The column “Value bounds” presents the range of values considered for each parameter during tuning. Additional details of the CNN model proposed for BIQA can be found in [17].

### 3.3. Development Tools and System Specifications

Our DNN model was developed in Python (version 3.10.14) using the following libraries (version numbers are provided in brackets):

- TensorFlow (v.2.10.1) for building and training models;
- NumPy (v.1.26.4) for numerical computations;
- Pandas (v.2.2.2) for data manipulation;
- Optuna (v.4.0.0) for hyperparameter tuning;
- Scipy (v.1.14.1) for scientific computing tasks;
- Matplotlib (v.3.9.2) for visualizations;
- OpenCV (v.4.10.0.84) for image processing.

We selected TensorFlow because of its comprehensive support for complex deep learning tasks. Another viable alternative is PyTorch (v.2.5.0) which is known for its dynamic computation graph and ease of use.

TensorFlow automatically handles GPU computations. However, a compatible version of the CUDA framework is required. The supported CUDA version, along with its associated components, is listed below.

- cuda-nvcc (v.12.4.131), a CUDA compiler;
- cudatoolkit (v.11.2.2), the CUDA framework;
- cudnn (v.8.1.0.77), a CUDA library providing highly optimized implementations of common deep learning procedures such as convolution, pooling, and normalization.

Each of the packages mentioned above was installed using Miniconda (v.24.7.1), a lightweight distribution of the Conda package manager.

Deep neural networks (DNNs) require powerful computing units. The computations were performed using a GPU and CUDA technology, which enables parallel computing. The computations were performed on a machine equipped with the following components: two Intel Xeon E5-2680 v2 processors (Intel, Santa Clara, CA, USA) operating at a frequency of 2.80 GHz, 48 GB of RAM, and an NVIDIA GeForce RTX 4070 graphics card (NVIDIA, Santa Clara, CA, USA) with 12 GB of memory.

## 4. Results

The evaluation of the proposed model requires testing on image databases. For this purpose, two publicly available and relatively recent databases were selected, each containing images with various distortions and their corresponding MOS values. The databases used are TID2013 [18] and KADID-10k [19]. Table 3 summarizes the key characteristics of these databases.

**Figure 2:**
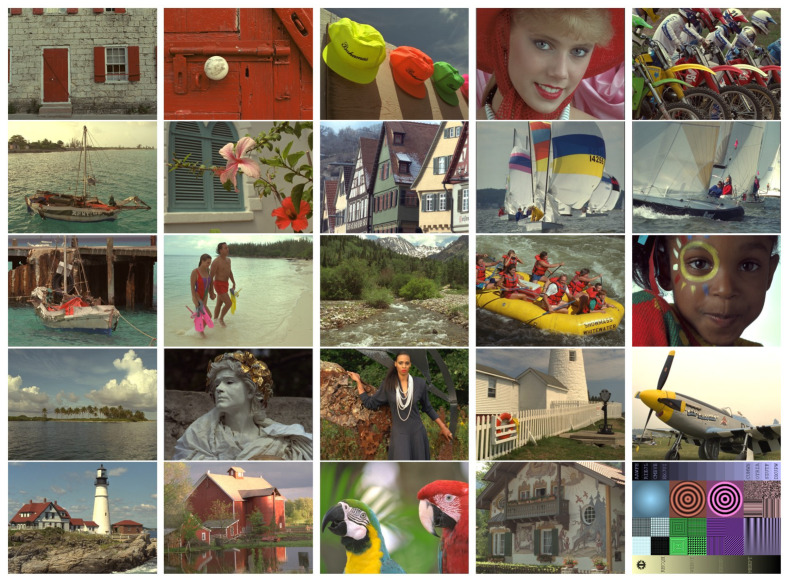
TID2013 (Tampere Image Database): reference images [18].

**Figure 3:**
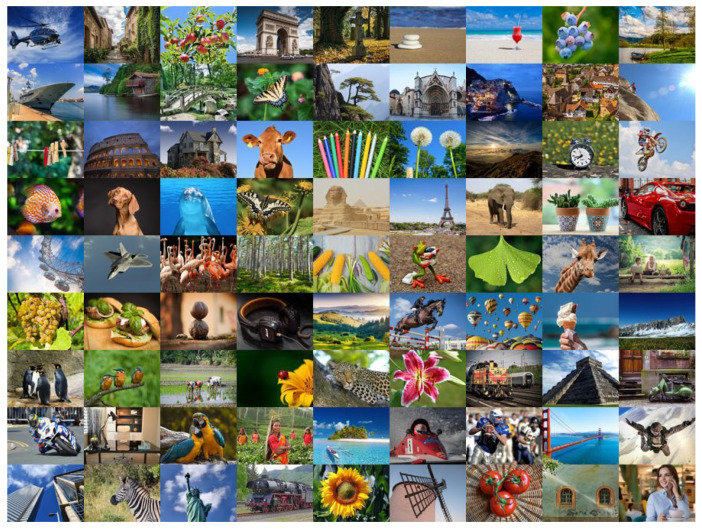
KADID-10k (Konstanz Artificially Distorted Image quality Database): reference images [19].

The TID2013 database consists of 25 reference images (Figure 2) that were subjected to 24 types of distortion at five different levels of severity. Notable examples among the 24 distortions include additive Gaussian noise, masked noise, JPEG compression, contrast variations, color quantization with dithering, and chromatic aberrations. Consequently, a total of 3000 images with subjective quality assessment (MOS) were generated. These quality scores were collected from human observers in a controlled laboratory environment. The KADID-10k database was created from 81 reference images (Figure 3), each of which was subjected to 25 types of distortion, with characteristics similar to those in TID2013, at five levels of severity. As a result, the database contains 10,125 distorted images. The database uses a differential mean opinion score (DMOS). The DMOS values for these images were obtained via crowdsourcing.

**Figure 4:**
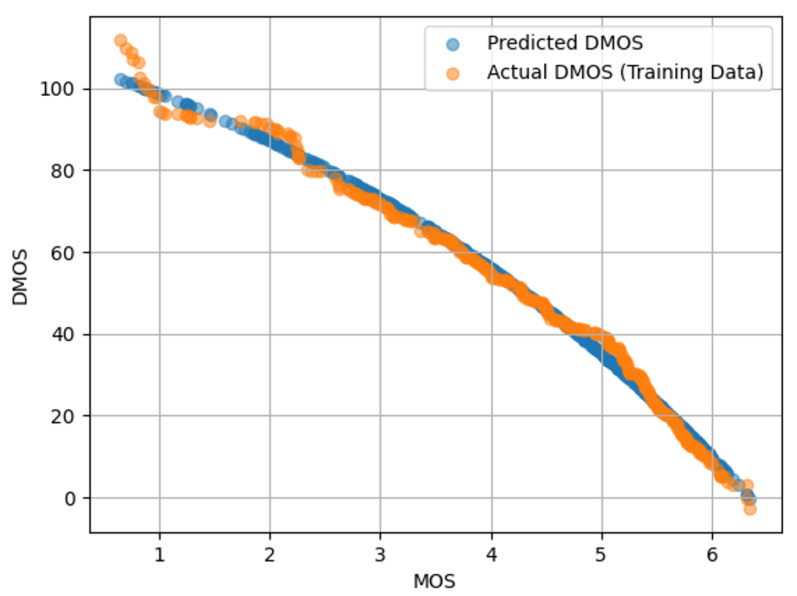
Scatter plots of subjective MOS versus IQA metrics obtained from the TID2013 database.

The databases use different scoring methods: DMOS ranges from 0 to 100, where 100 indicates the worst quality. In contrast, MOS ranges from 0 to 6, where 0 indicates the worst quality. To address these differences and ensure consistency, a logistic regression mapping was applied to align the quality metrics across the datasets. This step is essential when training a model in one dataset and evaluating it on another. An example of this mapping is presented in Figure 4.

Figure 4 shows a scatter plot with MOS on the x-axis and DMOS on the y-axis. The orange points represent the actual DMOS values from the training data used to fit the logistic regression model, while the blue points indicate the DMOS values predicted by the model. Although there is some divergence for low-quality images, the logistic regression adequately captures the nonlinear mapping between MOS and DMOS, enabling accurate transformation from one scale to the other.

In the field of BIQA, image quality metrics are typically compared against subjective perceptual ratings. For this purpose, Pearson’s linear correlation coefficient (PLCC), Spearman’s rank correlation coefficient (SROCC), and Kendall’s rank correlation coefficient (KROCC) are used. According to the recommendations outlined by Sheikh et al. [20], the calculation of PLCC should be preceded by a nonlinear regression based on a five-parameter logistic function:

(6) $$p\left(x,\beta \right)=\beta_{1}\left(\frac{1}{2}-\frac{1}{1+exp\left(\beta_{2}\left(x-\beta_{3}\right)\right)}\right)+\beta_{4}x+\beta_{5},$$

where $\beta_{i},i=1,\;2,\;\ldots ,$ 5—parameters, *x*—raw quality index.

The formulas for calculating the PLCC and SROCC correlation coefficients are as follows:

(7) $$PLCC=\frac{\sum_{i=1}^{N}\left(p_{i}-\bar{p}\right)\left(s_{i}-\bar{s}\right)}{\sqrt{\sum_{i=1}^{N}{\left(p_{i}-\bar{p}\right)}^{2}{\left(s_{i}-\bar{s}\right)}^{2}}},$$

where $p_{i}$ and $s_{i}$ represent the raw values of the subjective and objective measures, respectively, while $\bar{p}$ and $\bar{s}$ denote the mean values of the subjective and objective measures,

(8) $$SROCC=1-\frac{6\sum_{i=1}^{N}d_{i}^{2}}{N\left(N^{2}-1\right)},$$

where $d_{i}$ represents the difference between the ranks of two measures for the *i*-th observation, and *N* is the total number of observations. Higher correlation values indicate better BIQA performance. These two criteria evaluate prediction accuracy (PLCC) and prediction monotonicity (SROCC).

In the experiments described below, which compare different neural networks used in BIQA, we only report the values of the PLCC and SROCC indices, as they are the most commonly used metrics in BIQA research. Our comparison includes a set of well-known DNN-based solutions as well as newer methods that have recently gained significant attention in the research community due to their strong performance. These approaches represent a variety of architectural designs, including models based on transfer learning, dual-branch structures, multi-output strategies, and transformer-based mechanisms. The results for the compared networks were taken from [21].

Table 4 presents the results of our network trained and tested on the TID2013 database. The proposed model demonstrates strong performance, achieving a PLCC of 0.871 and an SROCC of 0.846, outperforming the DB-CNN and MEON models and closely approaching the state-of-the-art TReS network. Importantly, unlike many existing methods, our network does not rely on transfer learning from large pre-trained models or datasets, such as models trained on ImageNet (used by DB-CNN) or ResNet-50 (used by TReS). A comparison of the proposed solution with another lightweight NIMA [22] model based on the MobileNet architecture favors our model in terms of both correlation metrics and the number of parameters.

Furthermore, the architectures of these competing methods incorporate additional complex design elements, such as dual-output heads (MEON), dual-branch structures (DB-CNN), and transformers (TReS, DEIQT). In contrast, our model employs a significantly simpler architecture with fewer parameters and can be trained from scratch using standard, well-established CNN techniques, making it both efficient and accessible.

For further comparison, two additional models (ExIQA [27], CoDI-IQA [28]) from 2024 to 2025 were also included. The ExIQA model addresses the BIQA task from the perspective of distortion identification, aiming to determine both the types and the strengths of distortions in an image by leveraging a Vision–Language Model (VLM). The identified distortions are subsequently provided as input to a regressor in order to predict the image quality score. The training of ExIQA was conducted on a large-scale dataset containing over one hundred thousand multi-distorted images based on the KADID-10k database.

The CoDI model (Cross-domain Distortion Identification for Image Quality Assessment), similar to ExIQA, estimates image quality indirectly by analyzing distortion types and strengths. It leverages deep networks such as ResNet-50 (CNN) and Swin Transformer (ViT) as encoders to disentangle content-dependent and distortion-related features. Cross-dataset experiments demonstrate its strong generalization capability. Although both new models achieved very high PLCC and SROCC values across various test image databases, their training requires adjusting tens of millions of parameters.

Table 5 presents the results of the proposed method trained and evaluated on the KADID-10k database. The proposed model achieves a PLCC of 0.789 and an SROCC of 0.798, representing a notable drop in performance relative to the TID2013 results. Although the model outperforms MEON, it falls short of the other competing methods on this dataset. The model we propose performs worse than another lightweight model (GreenBIQA [29]), but the number of parameters is only half that of the other model.

**Figure 5:**
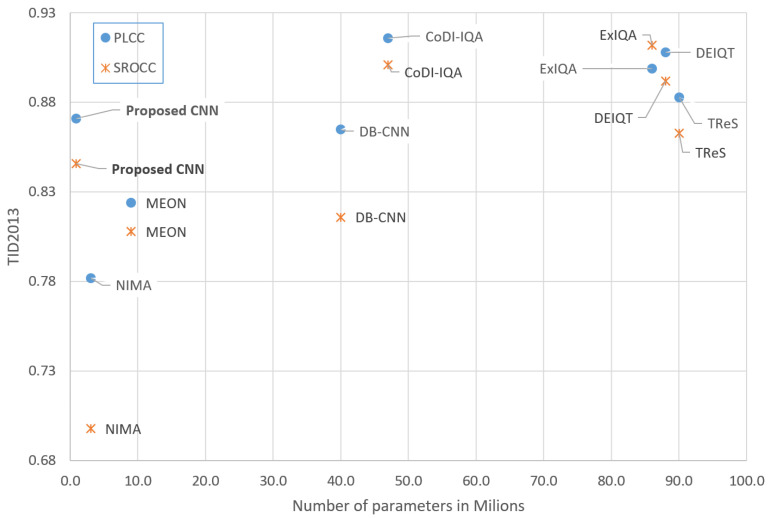
Correlation coefficients vs. number of parameters (TID2013).

**Figure 6:**
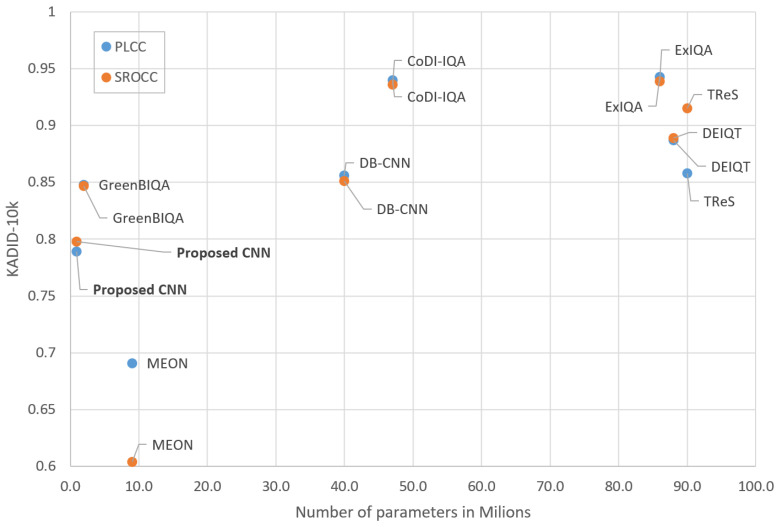
Correlation coefficients vs. number of parameters (KADID-10k).

Figure 5 and Figure 6 show the achieved PLCC and SROCC coefficients for all compared models as a function of the number their trainable parameters. The proposed architecture stands out by having the lowest number of trainable parameters while maintaining satisfactory correlation performance.

The performance deterioration on KADID-10k is notable compared to TID2013. This discrepancy can be attributed to the observation that hyperparameter tuning was conducted primarily on the TID2013 database, while extensive tuning on KADID-10k was not feasible due to computational limitations. Although such optimization could potentially improve the results, the training process requires several hours per configuration, making comprehensive hyperparameter exploration impractical given the available computational resources.

While the results obtained for our CNN model were not directly compared with those of Kang’s CNN, it is reasonable to assume that our model would outperform the Kang approach. Kang’s models were evaluated exclusively on the TID2008 database, which contains images subjected to a narrower and less diverse range of distortions compared to the TID2013 database. Consequently, our approach can be considered not only effective, but also efficient and accessible for practical applications.

Table 6 presents the durations of training and evaluation. The reported number of epochs refers exclusively to those actually used during training; epochs omitted due to early stopping are not included. The evaluation time corresponds to the total duration of image quality prediction on the left-out test set. As shown, training on KADID-10k required substantially more time and a greater number of epochs compared to TID2013. This outcome is expected, as KADID-10k contains a larger number of samples and therefore requires more iterations for the model to converge.

The low average prediction times achieved by the proposed BIQA model indicate not only a lightweight network architecture and efficient implementation but also demonstrate its potential for real-time operation and energy-efficient deployment—an aspect of particular importance for mobile devices and embedded systems. These prediction times should be considered in conjunction with the accuracy of the model, i.e., the reliability of quality assessments.

## 5. Conclusions

DNN-based solutions have become the standard in BIQA, with recent models increasingly relying on complex architectures, transfer learning, and attention mechanisms to enhance performance. Although these advances have yielded notable improvements, they often introduce drawbacks such as increased model size, higher training complexity, and a strong dependence on pre-trained networks. Our proposed method, inspired by the first CNN-based approaches in BIQA, revisits the principle of architectural simplicity without compromising effectiveness. We integrate modern machine learning techniques, including Bayesian hyperparameter optimization with Optuna, to strengthen performance while maintaining this classical foundation. Consequently, our model achieves performance comparable to current state-of-the-art solutions while remaining relatively lightweight, interpretable, and easy to train from scratch. This balance between simplicity and effectiveness makes it particularly suitable for future scenarios in which scalability, optimization, or deployment efficiency are essential.

## Figures and Tables

**Table 1:** Architecture details of the CNN model.

Layer	Output Shape	Info	Stride/Padding
Input	32×32×3	N/A	N/A
Conv2D	32×32×32	32 3×3 kernels	1/same
BatchNorm	32×32×32	N/A	–
MaxPooling	16×16×32	2×2 pool size	2/valid
Conv2D	16×16×64	64 3×3 kernels	1/same
BatchNorm	16×16×64	N/A	–
MaxPooling	8×8×64	2×2 pool size	2/valid
Conv2D	8×8×128	128 3×3 kernels	1/same
BatchNorm	8×8×128	N/A	–
MaxPooling	4×4×128	2×2 pool size	2/valid
Conv2D	4×4×256	256 3×3 kernels	1/same
BatchNorm	4×4×256	N/A	–
MaxPooling	2×2×256	2×2 pool size	2/valid
Flatten	1024×1	N/A	–
Dense	512×1	N/A	–
Dropout	512×1	rate = 0.5	–
Dense (Output)	1×1	N/A	–

**Table 2:** Hyperparameter value bounds.

Parameter	Value Bounds
n_neurons	[500, 1000]
eta	[0.001, 0.01]
dropout_rate	[0, 0.8]

**Table 3:** Summary of image quality assessment databases.

Database	Year	Originals	Distort.	Images
TID2013	2013	25	24	3000
KADID-10k	2019	81	25	10,125

**Table 4:** Performance comparison of IQA methods trained and tested on the TID2013 database.

Method	PLCC	SROCC	Param.	Year
DB-CNN [23]	0.865	0.816	∼40 M	2018
MEON [24]	0.824	0.808	∼9 M	2018
NIMA [22]	0.782	0.698	∼3 M	2018
TReS [25]	0.883	0.863	∼90 M	2022
DEIQT [26]	0.908	0.892	∼88 M	2023
ExIQA [27]	0.899	0.912	∼86 M	2024
CoDI-IQA [28]	0.916	0.901	∼47 M	2025
Proposed CNN	0.871	0.846	∼0.9 M	2025

**Table 5:** Performance comparison of IQA methods tested on KADID-10k.

Method	PLCC	SROCC	Param.	Year
DB-CNN [23]	0.856	0.851	∼40 M	2018
MEON [24]	0.691	0.604	∼9 M	2018
TReS [25]	0.858	0.915	∼90 M	2022
GreenBIQA [29]	0.848	0.847	∼2 M	2022
DEIQT [26]	0.887	0.889	∼88 M	2023
ExIQA [27]	0.943	0.939	∼86 M	2024
CoDI-IQA [28]	0.940	0.936	∼47 M	2025
Proposed CNN	0.789	0.798	∼0.9 M	2025

**Table 6:** Training and evaluation times for each database.

Database	No. of Epochs	Avg. Time per Epoch (min)	Evaluation Time (s)	Avg. Time per Image (s)
TID2013	24	3	23	0.038
KADID-10k	74	21	42	0.021

## Data Availability

Data are contained within the article.

## Abbreviations

The following abbreviations are used in this manuscript:

BIQA	Blind Image Quality Assessment
CNN	Convolutional Neural Network
CUDA	Compute Unified Device Architecture
DL	Deep Learning
DMOS	Differential Mean Opinion Score
DNN	Deep Neural Network
GAN	Generative Adversarial Network
GPU	Graphics Processing Unit
HVS	Human Visual System
IQA	Image Quality Assessment
KADID	Konstanz Artificially Distorted Image quality Database
KROCC	Kendall Rank Order Correlation Coefficient
MAE	Mean Average Error
MOS	Mean Opinion Score
NSS	Natural Scene Statistics
PLCC	Pearson Linear Correlation Coefficient
ReLU	Rectified Linear Unit
SROCC	Spearman Rank Order Correlation Coefficient
TID	Tampere Image Database
TPE	Tree-structured Parzen Estimator
